# Combination of (interferon beta-1b, lopinavir/ritonavir and ribavirin) versus favipiravir in hospitalized patients with non-critical COVID-19: A cohort study

**DOI:** 10.1371/journal.pone.0252984

**Published:** 2021-06-10

**Authors:** Areej A. Malhani, Mushira A. Enani, Fatemeh Saheb Sharif-Askari, Mona R. Alghareeb, Roaa T. Bin-Brikan, Safar A. AlShahrani, Rabih Halwani, Imad M. Tleyjeh

**Affiliations:** 1 Clinical Pharmacy Department, Pharmacy Services Administration, King Fahad Medical City, Riyadh, Saudi Arabia; 2 Infectious Diseases Section, Department of Medical Specialties, King Fahad Medical City, Riyadh, Saudi Arabia; 3 Sharjah Medical Institute of Research, College of Medicine, University of Sharjah, Sharjah, United Arab Emirates; 4 Clinical Research Coordinator, Collage of Medicine, King Fahad Medical City, Riyadh, Saudi Arabia; 5 Outpatient Pharmacy Department, Pharmacy Services Administration, King Fahad Medical City, Riyadh, Saudi Arabia; 6 Department of Clinical Sciences, College of Medicine, University of Sharjah, Sharjah, United Arab Emirates; 7 Prince Abdullah Ben Khaled Celiac Disease Chair, Department of Pediatrics, Faculty of Medicine, King Saud University, Saudi Arabia; 8 College of Medicine, Alfaisal University, Riyadh, Saudi Arabia; 9 Division of Infectious Diseases, Mayo Clinic College of Medicine and Science, Rochester, Minnesota, United States of America; 10 Division of Epidemiology, Mayo Clinic College of Medicine and Science, Rochester, Minnesota, United States of America; Azienda Ospedaliero Universitaria Careggi, ITALY

## Abstract

**Objectives:**

Our study aims at comparing the efficacy and safety of IFN-based therapy (lopinavir/ritonavir, ribavirin, and interferon β-1b) vs. favipiravir (FPV) in a cohort of hospitalized patients with non-critical COVID-19.

**Methods:**

Single center observational study comparing IFN-based therapy (interferon β-1b, ribavirin, and lopinavir/ritonavir) vs. FPV in non-critical hospitalized COVID-19 patients. Allocation to either treatment group was non-random but based on changes to national treatment protocols rather than physicians’ selection (quasi-experimental). We examined the association between IFN-based therapy and 28-day mortality using Cox regression model with treatment as a time-dependent covariate.

**Results:**

The study cohort included 222 patients, of whom 68 (28%) received IFN-based therapy. Antiviral therapy was started at a median of 5 days (3–6 days) from symptoms onset in the IFN group vs. 6 days (4–7 days) for the FPV group, *P* <0.0001. IFN-based therapy was associated with a lower 28-day mortality as compared to FPV (6 (9%) vs. 18 (12%)), adjusted hazard ratio [aHR] (95% Cl) = 0.27 (0.08–0.88)). No difference in hospitalization duration between the 2 groups, 9 (7–14) days vs. 9 (7–13) days, P = 0.732 was found. IFN treated group required less use of systemic corticosteroids (57%) as compared to FPV (77%), P = 0.005 after adjusting for disease severity and other confounders. Patients in the IFN treated group were more likely to have nausea and diarrhea as compared to FPV group (13%) vs. (3%), P = 0.013 and (18%) vs. (3%), P<0.0001, respectively.

**Conclusion:**

Early IFN-based triple therapy was associated with lower 28-days mortality as compared to FPV.

## Introduction

The pandemic of severe acute respiratory syndrome coronavirus-2 (SARS-CoV-2) has resulted in more than 100 million cases and over 2 million deaths worldwide as of February 16, 2021 [1]. Despite the huge global death toll, there is a paucity of approved treatment options which include the antiviral medication remdesivir with or without baricitinib, and dexamethasone. Other potential antiviral treatments such as favipiravir (FPV) and interferon (IFN) based therapy have had conflicting results in randomized controlled trials (RCTs) and are not currently endorsed for COVID-19 treatment by medical societies [2].

Type I IFNs, first discovered in 1957, are well known cytokines which play an important role in both innate and cell-mediated immunity against viral infections. IFNs antiviral effect is achieved through the activation of interferon-stimulated genes (ISGs) which encode protein products that work alone or in combinations to achieve one or more cellular outcomes, including antiproliferative activities, antiviral defense, and stimulation of adaptive immunity [3]. Because of the importance of endogenous IFNs in early viral infections control, there has been several studies examining its use in therapeutic regimens for coronavirus infections as early as 2004 [4, 5].

Moreover, other antivirals have been examined in COVID-19. The protease inhibitor lopinavir/ritonavir (LPV/r), demonstrated inhibition of MERS-CoV and SARS-CoV replication in vitro. Early during the pandemic, the available data for the use of LPV/r in COVID-19 was controversial and varied from studies with clearly negative results to more favorable efficacy.

Early RCTs [6–8] reports on the management of COVID-19 in-patients with combination LPV/r or placebo did not show a beneficial effect on mortality or the need for mechanical ventilation. Subsequently, the SOLIDARITY trial was stopped due to preliminary results showing that LPV/r produce no reduction in the mortality of COVID-19 hospitalized patients when compared to standard of care [9]. Second, ribavirin (RBV), a guanosine analogue and a broad-spectrum antiviral drug, has been evaluated in patients with MERS-CoV and SARS-CoV and later had high half maximal effective concentration (EC50) of 109.50 μM and selectivity index against SARS-CoV-2 in Vero cells [10].

One of the first RCTs conducted during the pandemic was a phase 2 multicenter trial in hospitalized adult patients with COVID-19 in Hong Kong. Patients were allocated to a 14-days combination of LPV/r, ribavirin (RBV) and IFN β-1b (triple combination therapy) vs. 14 days of LPV/r. Triple antiviral therapy was more effective in comparison to LPV/r in symptoms resolution, duration of viral shedding and length of hospitalization in patients with mild to moderate COVID-19 [11].

Another antiviral drug, FPV, a competitive inhibitor of RNA-dependent RNA polymerase (RdRp), which was first approved in Japan for treating influenza, has also been examined in COVID-19. The US Food and Drug Administration has approved it as an investigational new drug to proceed with an expanded phase 2 clinical trial for COVID-19. Experimental studies have demonstrated that FPV can achieve an effective antiviral concentration against the SARS-CoV-2 virus within a safe therapeutic dose. Many countries have recommended the usage of FPV therapy in mild to moderate COVID-19 such as Japan, India, Russia, Thailand, Kenya and Saudi Arabia [12, 13]. However, a recent systematic review by the EUnetHTA Rolling Collaborative Review Authoring Team showed that the existing evidence does not support the use of FPV as monotherapy or combination therapy for COVID-19 [14].

To the best of our knowledge, there is no study to date comparing FPV to IFN-based regimens in the treatment of COVID-19. Therefore, we aimed to evaluate the safety and efficacy of triple combination regimen of LPV/r, RBV and IFN β-1b, in comparison with FPV in the treatment of patients hospitalized with mild, moderate, and severe COVID-19 using an observational cohort study design, where treatment allocation was based on changes to national treatment protocols rather than physicians’ selection (quasi-experimental).

## Materials and methods

### Study setting

**Fig 1:**
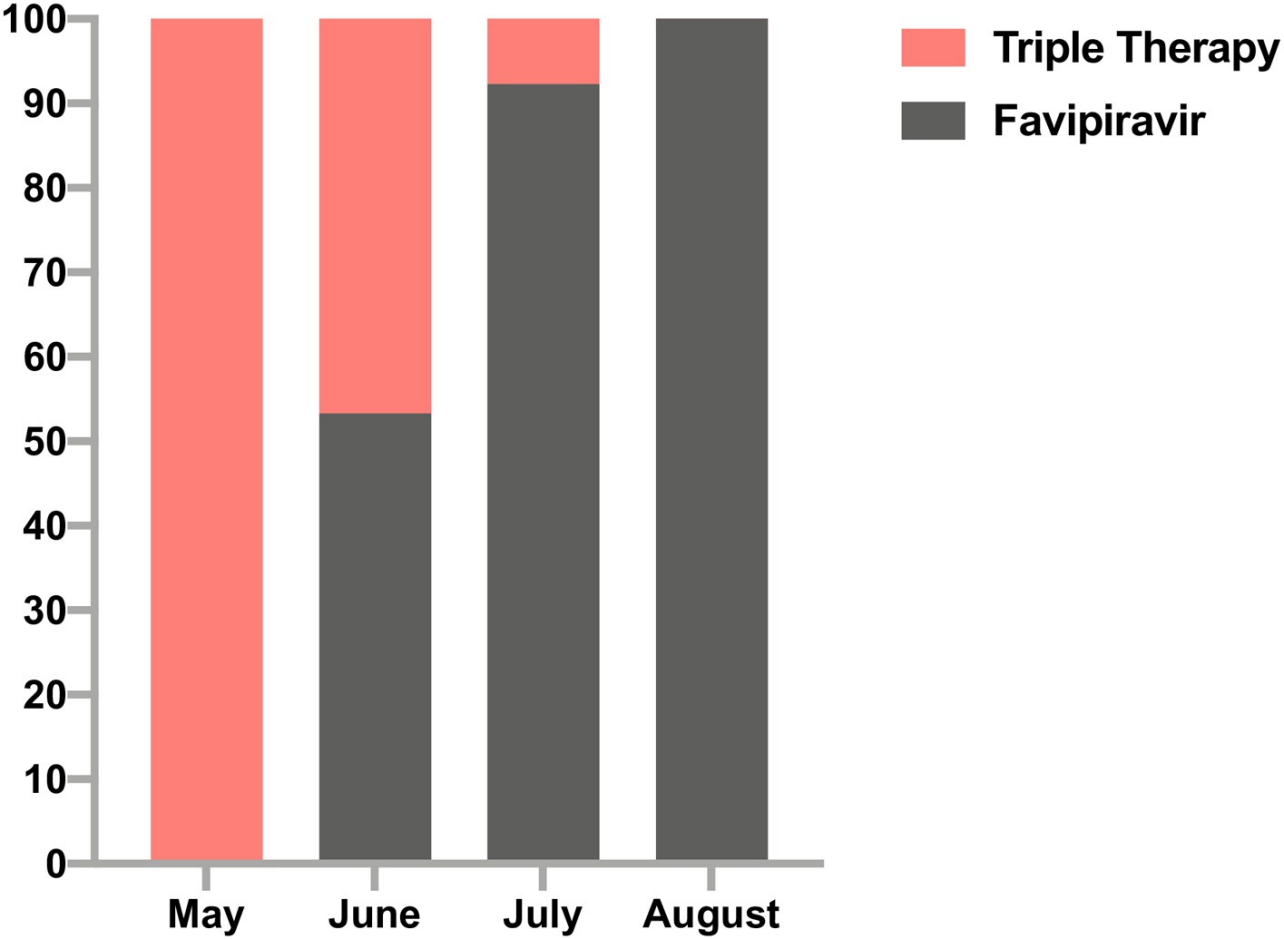
Distribution of treatment modalities over time.

Our study is a quasi-experimental comparative study of a prospective cohort of patients with mild to severe COVID-19 that were admitted to King Fahad Medial City (KFMC), the largest tertiary and teaching hospital under the Ministry of Health (MOH), in Saudi Arabia. KFMC is a Joint Commission International (JCI) accredited hospital, has been assigned by the MOH as a COVID-19 community treating center for the central region of Saudi Arabia. Allocation of patients to FPV or triple IFN-based combination regimen was non-random but based on a “quasi-experimental” natural experiment design due to the introduction of FPV in Saudi national COVID-19 treatment guidelines and its availability at KFMC by mid-June 2020. Neither physicians nor patients influenced the allocation of patients to treatment groups except within the context of the institutional treatment protocol. Triple IFN-based combination regimen was used prior to mid-June 2020 whereas FPV was used later on (Fig 1). KFMC Institutional Review Board (IRB) approved the study and waived the need for informed consent.

### Patient selection

The study cohort included consecutive COVID-19 patients ≥18 years old who were hospitalized at KFMC between May 1st and September 30, 2020. Confirmation of infection with SARS-CoV-2 was made by nasopharyngeal swab polymerase chain reaction (PCR). These patients should have also received either triple combination therapy or FPV. We included patients hospitalized with mild, moderate, or severe disease as per the National Institute of Health (NIH) classification [15]. Critical illness was considered as one of the study endpoints. We did not include pre-hospitalization ambulatory management data as these were not available to us. Most patients received only supportive over the counter medications before hospital admission while a few received hydroxychloroquine in the Ministry of Health Primary Care Centers.

### Drug therapy

KFMC COVID-19 management protocol for the treatment of COVID-19 infection was followed for treatment options (S1 Table). This treatment protocol was prepared by KFMC COVID-19 scientific taskforce, a multidisciplinary team of infectious diseases, intensive care physicians, and clinical pharmacists. Our treatment protocol included: a 14-day course of LPV/r 400mg/100 mg twice daily, RBV 400 mg twice daily and zero to three doses of received recombinant IFN beta-1b (Betaferon, Bayer) as a subcutaneous injection (at a dose of 0.25 mg [8 million IU] in 1 ml of solvent) on alternate days 1, 3 and 5 (depending on the number of days between admission and symptoms onset) [11], or a 7 to 10-days course of FPV regimen 1800 mg/dose twice daily on day one, followed by 800 mg/dose twice daily for 7–10 days. Interferon-based therapy was not recommended beyond 7 days of symptoms onset. Remdesivir was not available in Saudi Arabia. Criteria for steroids use (methylprednisolone 0.5–1 mg/kg/day divided in two doses for five days) or dexamethasone 6 mg orally (once daily or equivalent intravenous dose for 10 days) included for moderate COVID-19 patients who are at need for supplemental oxygen ≥ 4 L or increasing oxygen requirements from the baseline and for the mechanically ventilated severe COVID-19 patients with acute respiratory distress syndrome (ARDS). Tocilizumab 8mg/kg was considered in patients with cytokine release syndrome (CRS), rapidly worsening respiratory symptoms/signs, and absence of systemic bacterial or fungal coinfection in addition to 2 or more of the following criteria: a) ferritin >300 ug/L doubled within 24 hours, b) ferritin >600 ug/L at presentation, c) lactic acid dehydrogenase (LDH) >250, d) increased D-dimer (>1 mcg/mL), e) C-reactive protein (CRP) > 70 mg/L. Interlukin-6 (IL-6) assay was not available. All patients received standard of care management in addition to the previous therapeutic options.

### Data collection

Patients were evaluated daily by infectious disease physicians and data were documented prospectively in the daily assessment form (S1 Table). Additional data were retrieved from patients’ electronic medical records. To assess the safety profile of IFN-based regimen, serial levels of hemoglobin, platelet count, alanine aminotransferase (ALT), and bilirubin were evaluated. The study outcomes were ascertained at 28 days after admission irrespective of hospital discharge. For patients who were discharged before 28 days, the research team called patients or their families more than once: 1) to ask about side effect profiles if they were still receiving the treatment regimen, and 2) to ascertain vital status and re-hospitalizations.

### Outcomes

The study primary outcome was all-cause mortality, and the secondary outcomes were the need of mechanical ventilation or intensive care unit admission at day 28 after hospital admission. We have also evaluated the following outcomes: a) National Early Warning Score (NEWS-2) at day 1, 3 and 7 of hospitalization, b) use of steroids, c) use of tocilizumab within 28 days. Safety related endpoints included: patient reports of adverse events, and levels of hemoglobin, platelet count, alanine aminotransferase (ALT), and bilirubin at day 1, 3 and 7 of hospitalization. The NEWS-2 is an aggregate scoring system predicting diseases severity and mortality [16].

### Statistical analysis

Chai-square or Fisher exact tests for categorical variables and Student *t* test or Mann-Whitney *U* test for continuous variables (where appropriate) were used to compare baseline clinical characteristics and interventions of patients who received triple therapy to those who did not.

For the primary outcome of 28-day mortality and secondary outcomes of mechanical ventilation or intensive care unit admission, a sequential series of two Cox proportional hazard models were developed. Time zero was considered to be the hospital admission date in all survival analyses. Model 1 was developed by adding patient baseline variables at admission (age, gender, body mass index, and SpO2 value) and Model 2 was developed by adding patient variables related to COVID-19 severity (serum CRP, ferritin, D-Dimer, and bilirubin levels, WBC), oxygen needs and use of systemic corticosteroids during hospital stay [17] (S2–S4 Tables). The presence of collinearity among variables was examined by the evaluation of variance inflation factors. Model improvement through addition of variables were assessed using measures of discrimination and goodness of fit [18]. Concordance statistics (C statistics) is a measure of discrimination which refers to the ability of a model to distinguish between two groups of outcomes [19]. Overall model fit for sequential Cox proportional hazard models was measured using the Akaike Information Criterion (AIC), which takes into account both model statistical goodness of fit (-2log-likelihood) and the number of estimated variables used to develop the model by imposing a penalty for increasing the number of variables in the model [20–22]. Higher values for C statistic and lower values for AIC indicate better models. Moreover, triple IFN-based therapy was considered as a time-varying covariate to avoid “immortal time bias” or “survivor selection bias”, which occurs because patients who live longer are more likely to receive treatment than those who die early [23–25]. In this study since time from symptoms onset to start of treatment was different for individual patients, triple IFN-based therapy was considered as a time-varying covariate in the model. The hazard ratio with 95% CIs from the best Cox regression model (model 2) was used to estimate the mortality ratio of IFN based therapy vs. FPV therapy at 28-day follow up. Kaplan–Meier survival curves were constructed to show cumulative survival over the 28-day period.

We also compared NEWS-2 score at day 1, 3 and 7 of hospitalization, length of hospital stay, and the use of supportive treatments for COVID-19 such steroids and tocilizumab between IFN based therapy vs. FPV therapy groups. For evaluating NEWS-2 score between these two treatment groups at the given days, we have used generalized linear mixed models which included fixed effect of treatment and random effect of age, male sex, body mass index, and time from start of symptoms onset to start of treatment, days.

Furthermore, for assessing the use of supportive treatments between IFN based therapy vs. FPV therapy groups logistic regression models have been developed and adjusted for both patient baseline variables at admission and patient COVID-19 related severity variables during hospital stay.

All tests were 2 tailed and a P value of less than 0.05 was considered statistically significant. Statistical analysis was performed using SPSS (version 26.0), R software (version 3.6.1) and PRISM (version 8). A file consisting of all patient’s parameters used in the analysis is provided in S1 File.

## Results

### Baseline clinical characteristics

Of the 243 COVID-19 patients, 21 patients were excluded from the study because they received dual LPV/r and RBV without IFN as they presented one week after symptoms onset. 222 patients were included in the final analysis, of whom 68 (28%) received IFN-based therapy. No statistically significant differences in age, sex, body mass index and comorbidities were observed among the 2 groups. Patients who treated with IFN-based therapy were more likely to be admitted with severe disease (45% vs 32%; *P* = 0.067) or mild disease (10% vs 22%; *P* = 0.04), with more fever and gastrointestinal symptoms (Table 1). Time from symptoms onset to hospitalization was 4 days (3–5.5 days) for the IFN-based therapy group vs. 5 days (3–7 days) for the FPV group, *P* = 0.022. Antiviral therapy was commenced at a median of 5 days (3–6 days) from symptoms onset in the IFN-based therapy group vs. 6 days (4–7 days) for the FPV group, *P* <0.0001. The distribution of triple therapy vs. FPV over time (May-June 2020) confirmed that allocation of patients to the 2 treatment groups was due to treatment protocol changes and therefore the study was less susceptible to selection bias or confounding by indication (Fig 1).

**Table 1:** Baseline clinical characteristics of the study population.

	IFN-based therapy (n = 68)	Favipiravir (n = 154)	*P-value*
Age, years, mean (SD)	52 (14)	55 (14)	0.214
Sex			
Men	51 (75)	101 (66)	0.210
Women	17 (25)	53 (34)	
BMI, mean (SD)	29.59 (6)	29.79 (7)	0.867
Time from start of symptoms onset to start of treatment, days	5 (3–6)	6 (4–7)	<0.0001
Time from start of symptoms onset to hospital admission, days	4 (3–5.5)	5 (3–7)	0.022
COVID-19 severity			
Severe	30 (45)	48 (32)	0.067
Moderate	30 (45)	70 (46)	0.884
Mild	7 (10)	34 (22)	0.040
**Underlying comorbidities**			
Diabetes	35 (51)	79 (51)	1.000
Hypertension	31 (46)	74 (48)	0.772
Coronary artery disease	12 (18)	14 (9)	0.074
Cerebrovascular disease	0	4 (3)	0.315
Thyroid disease	5 (7)	10 (6)	0.776
Chronic hepatitis C	0	1 (1)	1.000
Chronic kidney disease	0	3 (2)	0.555
Malignancy	1 (2)	3 (2)	1.000
Smoker	4 (6)	5 (3)	0.252
**Symptoms and signs**			
Fever	55 (81)	89 (60)	0.001
Chills	2 (3)	0 (2)	0.093
Cough	51 (75)	119 (77)	0.733
Sputum	2 (3)	1 (1)	0.223
Shortness of breath	60 (88)	124 (80)	0.181
Sore throat	4 (6)	3 (2)	0.205
Myalgia	5 (7)	4 (3)	0.137
Malaise	2 (3)	3 (2)	0.643
Nausea or vomiting	9 (13)	5 (3)	0.013
Diarrhea	12 (18)	5 (3)	<0.0001
Rhinorrhea	3 (4)	0	0.028
Anosmia	4 (6)	1 (1)	0.032
Headache	9 (13)	1 (1)	<0.0001
Chest tightness	9 (13)	3 (2)	0.001
Anorexia	6 (9)	2 (2)	0.011
**Baseline laboratory findings (normal range)**			
Heamoglobin (11–16 g/dL)	13.6 (12–14.3)	13.5 (12.1–14.7)	0.995
White cell count (3.9–11.10 x 10^9^ per L)	6.3 (4.8–9.1)	7.2 (5.2–10.2)	0.205
Neutrophils (1.35–7.5 x 10^9^ per L)	5 (3–8.3)	5.2 (3.8–8.5)	0.513
Lymphocytes (1.5–4.3 x 10^9^ per L)	0.93 (0.69–1.40)	0.98 (0.75–1.45)	0.484
Platelets (115–435 x 10^9^ per L)	208 (155.5–298.5)	229 (173–284)	0.562
Alanine transaminase (0–55 U/L)	35 (25–60)	37 (24–60.5)	0.849
Aspartate transaminase (5–34 U/L)	51.5 (33–72)	44 (32–65)	0.583
Alkaline phosphatase (40–150 U/L)	72.5 (55–97)	79 (59–106)	0.246
Lactate dehydrogenase (125–220 U/L)	488 (353–696.5)	493 (363–639)	0.831
Bilirubin (3–20 μmol/L)	4.9 (3.7–7.6)	4.4 (3.3–6.4)	0.283
Creatinine (49–90 μmol/L)	72 (61–88.5)	75 (63–98)	0.363
Urea (3.5–7.2 mmol/L)	4.3 (3.1–6.1)	4.7 (3.6–7.7)	0.056
Creatinine Kinase (29–168 U/L)	187 (85–398)	122 (51–264)	0.177
C-reactive protein (1.0–3.0 mg/L)	117.5 (73.7–145)	105 (44.7–161)	0.581
Erythrocyte sedimentation	91 (67–97)	73.5 (47–99)	0.456
Ferritin (10–204 ng/mL)	712.4 (299.2–1462.4)	637.9 (235.4–1682.2)	0.694
D Dimer (0–0.5 μ/mL)	0.60 (0.41–1.16)	0.75 (0.45–1.52)	0.143
Troponin (0–15.6 ng/L)	7.9 (2–19.2)	7.3 (2–18.6)	0.781
**Baseline radiological findings**			
Abnormal chest x-ray	65 (96)	143 (93)	0.559
Bilateral chest infiltrate	63 (93)	143 (93)	0.826

Data are n (%) or median (IQR). In the triple therapy group 68 patients were treated with triple combination of interferon beta-1b, lopinavir–ritonavir, and ribavirin. U/L = units per L.

### Efficacy outcomes

**Fig 2:**
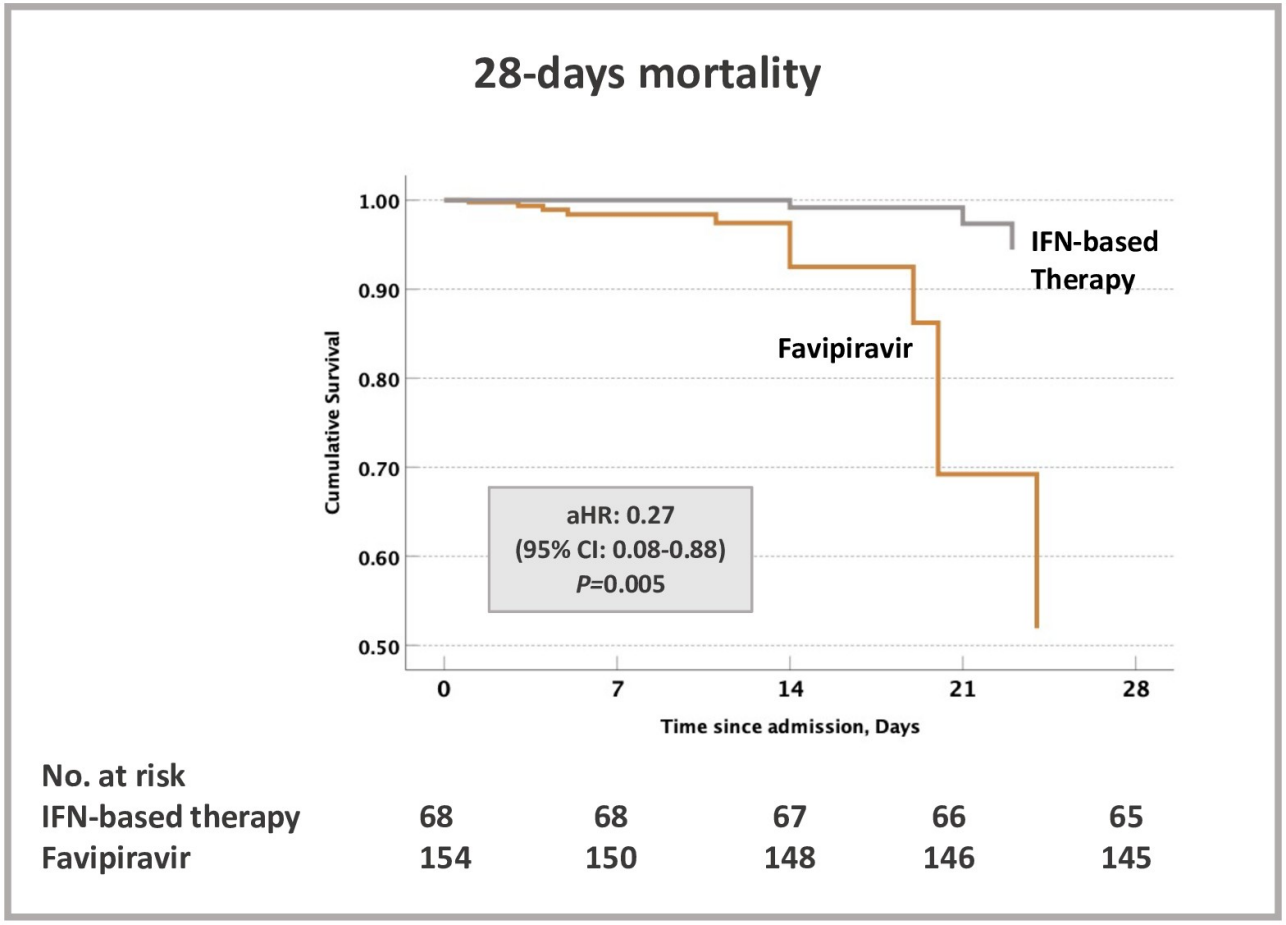
Kaplan–Meier curves for survival at 28 days follow up. Cox proportional model has been adjusted for both patient baseline variables at admission (patients’ age, male sex, body mass index, SpO2) and patient COVID-19 related severity variables during hospital stay (serum CRP, ferritin, and D-Dimer, and bilirubin levels, WBC, oxygen needs and systemic use of dexamethasone).

**Fig 3:**
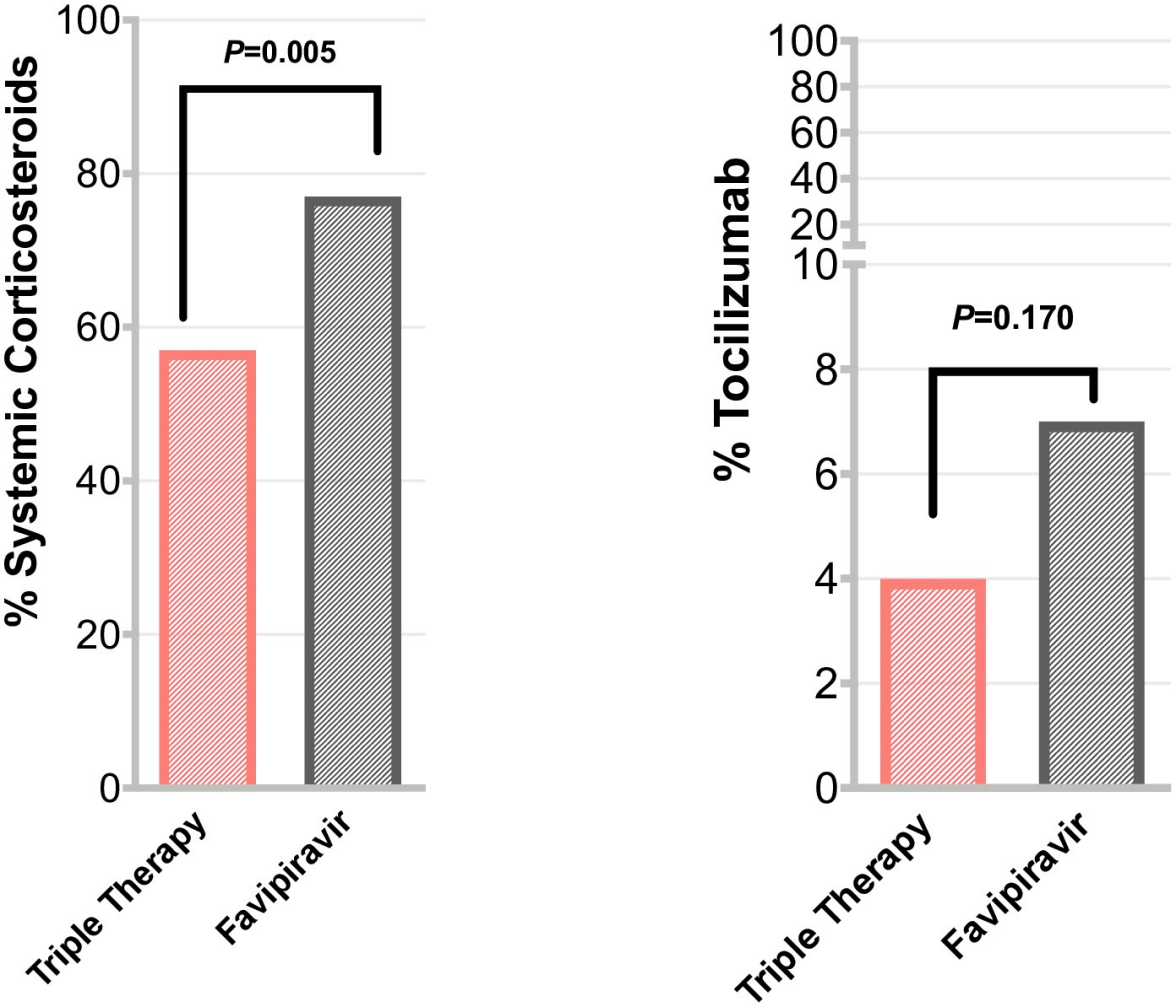
Comparison of proportions of patients requiring corticosteroids or tocilizumab during their treatment course in the interferon-based triple therapy vs. favipiravir groups*. *The use of supportive treatments such as tocilizumab and systemic corticosteroids between IFN-based therapy vs. FPV therapy groups was evaluated by logistic regression models adjusted for both patient baseline variables at admission (patients’ age, male sex, body mass index, SpO2) and patient COVID-19 related severity variables during hospital stay (serum CRP, ferritin, and D-Dimer, and bilirubin levels, WBC, and oxygen needs).

Favipiravir was associated with higher mortality as compared to those who received IFN-based therapy (Fig 2). IFN-based therapy was associated with a lower 28-day mortality (6 (9%) vs. 18 (12%)), adjusted hazard ratio [aHR] (95% Cl) = 0.27 (0.08–0.88). Of note, all the death in IFN-based and the majority in FPV (15 out of 18 death, 83%) occurred in severe COVID-19 patients (Table 2). No statistically significant difference in ICU admission or the need for mechanical ventilation between the 2 groups was observed (Table 2). IFN-based therapy group had lower NEWS2 score at day 3 and 7 as compared to FPV (Table 3). There was no difference in hospitalization duration between the 2 groups 9 (7–14) days vs. 9 (7–13) days, P = 0.732. After controlling for between group differences, IFN-based therapy group required less use of systemic corticosteroids (57%) during hospitalization as compared to FPV (77%), (P = 0.005, logistic regression) and Tocilizumab (4%) vs. (7%), (P = 0.170, logistic regression) (Table 3 and Fig 3).

**Table 2:** Primary and secondary outcomes.

Outcome	IFN-based therapy (n = 68)	Favipiravir (n = 154)	* HR (95% Cl)
**Primary outcome**			
Mortality at 28 days	6 (9%)	18 (12%)	0.27 (0.08–0.88)
Death by COVID-19 severity			
Severe	6 (100)	15 (83)	0.546
Moderate	0	2 (11)	1
Mild	0	1 (6)	1
**Secondary outcomes**			
Invasive mechanical ventilation	15 (22)	24 (16)	1.22 (0.68–2.20)
Intensive care unit admission	18 (26)	24 (16)	1.47 (0.86–2.51)

* Cox proportional model has been adjusted for both patient baseline variables at admission (patients’ age, male sex, body mass index, SpO2) and patient COVID-19 related severity variables during hospital stay (serum CRP, ferritin, D-Dimer, and bilirubin levels, WBC, oxygen needs and systemic use of dexamethasone).

Immortal time bias was accounted for in the Cox proportional model by considering medications as time-dependent variables.

**Table 3:** Temporal outcomes of interferon-based triple therapy vs. favipiravir.

	IFN-based therapy (n = 68)	Favipiravir (n = 154)	*P-value*
NEWS2, mean (95% CI)*			
Baseline	4.51 (4.00–5.02)	4.61 (4.29–4.92)	0.626
Day 3	2.64 (2.26–3.02)	3.35 (3.06–3.64)	0.008
Day 7	2.47 (2.16–2.77)	2.90 (2.61–3.20)	0.113
Duration of hospital stay, day*	9 (7–14)	9 (7–13)	0.732
**Need for Supportive Medications**#			
Systemic corticosteroids	39 (57)	118 (77)	0.005
Tocilizumab	3 (4)	10 (7)	0.170
Enoxaparin	51 (75)	127 (83)	0.056
Azithromycin	54 (79)	114 (74)	0.284
^a^Antibiotics	56 (82)	139 (90)	0.003

Data are n (%) or mean (%95 CI). In the triple therapy group 68 patients were treated with triple combination of interferon beta-1b, lopinavir–ritonavir, and ribavirin.

*Generalized linear mixed models were used which included fixed effect of treatment and random effect of age, male sex, body mass index, and time from start of symptoms onset to start of treatment, days.

^#^ Logistic regression models have been adjusted for both patient baseline variables at admission (patients’ age, male sex, body mass index, SpO2) and patient COVID-19 related severity variables during hospital stay (serum CRP, ferritin, D-Dimer, and bilirubin levels, WBC, and oxygen needs).

^a^Antibiotics defined as administration of any of: cefepime, ceftriaxone, vancomycin, or piperacillin/tazobactam.

### Safety endpoints

No differences between the 2 groups over time in hemoglobin, platelet count, or, ALT was found (S5 Table). There was a statistically significant, but clinically non-significant, increase in bilirubin level in the IFN-based therapy group. In addition, patients in the IFN-based therapy were more likely to have nausea and diarrhea as compared to FPV group (13%) vs. (3%), P = 0.013 and (18%) vs. (3%), P<0.0001, respectively.

## Discussion

### Findings

In this cohort study of patients hospitalized with non-critical COVID-19, we observed that: First, IFN-based triple therapy (IFN-β-1b plus RBV, plus LPV/r) was associated with lower 28-day mortality as compared to FPV; aHR 0.27, 95% CI (0.08–0.88). Second, IFN-based triple therapy treated patients had lower (NEWS2) score, currently recommended in the United Kingdom for risk stratification of COVID-19 outcomes, at days 3 and 7 of follow-up. Third, IFN-based triple therapy was associated with less need for systemic corticosteroids as an immunomodulatory therapy. Fourth, a clinically significant higher risk of serious adverse events was not observed with IFN-based triple therapy.

### Mechanisms

Type I IFN-signaling is essential for antiviral innate immunity, and plays a critical role in preventing the progression of viral infection and development of severe disease [26, 27]. In fact, Yuan et al. examined the antiviral activity of IFNs (IFN β-1a, IFN β-1b, pegylated IFN α-2a and IFN γ-1b) and other antivirals (lopinavir and remdesivir) using the plaque reduction assay. Among IFNs, IFN β-1b (Betaferon) was the most potent anti-SARS-CoV-2 with the lowest EC50 (31.2 IU/mL) and the highest selectivity index (>1602.6). The EC50 values for remdesivir and lopinavir were 1.04 and 11.6 μM, respectively [28].

Interestingly, gene variants affecting Type I IFN production and/or signaling have been shown to predispose patients to the development of severe COVID19 disease [29]. It is suggested that low levels of type I IFN, especially early on during infection, may result in an improper control of viral replication leading to tissue damage and exaggerated immune responses [30, 31]. Unlike SARS-CoV and MERS-CoV diseases where viral load peaks around days 7–10 after symptoms onset, viral load of SARS-CoV-2 reach its peaks at the time of symptom onset, indicating uncontrolled viral replication [32]. Therefore, early initiation of antiviral therapy is essential in COVID-19.

### Comparison to other studies

To date, there is no comparative study of IFN-based therapy vs. FPV for COVID-19. However, the therapeutic role of type I IFN for coronavirus infections has been investigated using both preclinical and clinical approaches. For example, an Italian experimental preclinical study has investigated the efficacy of IFN-β-1a controlling SARS-CoV-2 replication in Vero E6 cells. High concentrations of IFN-β-1a (5000 to 50 IU/mL) prevented SARS-CoV-2 replication in vero E6 cells while lower concentrations (0.05 and 0.01 IU/mL) had no effect on virus growth [33].

SARS-CoV expresses three IFN antagonistic proteins, open reading frame (ORF) 3b, ORF6, and N proteins that hinder different aspects of the interferon response. These proteins inhibit both IFN production and signaling [34]. Sallard et al., hypothesized that SARS-CoV-2 Orf6 and Orf3b proteins may have lost their anti-IFN functions which may elucidate the substantial sensitivity of SARS-CoV-2 to IFN-α treatment compared to other coronaviruses [35].

Nevertheless, there are few RCTs that examined the efficacy of IFN-based therapy vs. placebo or standard of care. Hung et al. performed the first prospective multicenter, randomized, phase 2 clinical trial on 127 hospitalized adult patients with mild to moderate COVID-19. They compared early initiation of 14-day combination of LPV/r, RBV, and three doses of IFN-β-1b (combination group) to 14 days of LPV/r (control group). The combination group had a significantly shorter median time to negative SARS-CoV-2 RT-PCR (7 days) compared to the control group (12 days); HR 4·37 [95% CI 1·86–10·24], p = 0·0010); and a significantly shorter time (4 days) to complete symptoms resolution, suppression of IL-6 levels, and a shorter hospital stay. However, the triple therapy group at baseline had significantly lower NEWS2 score than the control on day 1–7 which may have contributed to a favorable outcome in triple therapy cohort [11]. In another RCT of 92 patients, 42 patients received IFN β-1a in addition to the national protocol medications (hydroxychloroquine plus LPV/r or atazanavir-ritonavir). IFN-β-1a (44-μg/ml (12 million IU/ml)) was injected subcutaneously three times a week for two consecutive weeks. The control group of 39 patients were treated by the national protocol medications only. On day 14, 66.7% patients in the IFN group and 43.6% of patients in the control group were discharged (odds ratio [OR], 2.5; 95% confidence interval [CI], 1.05–6.37). A statistically significant lower mortality in the IFN than the control group (19% versus 43.6%) at day 28. Early administration of IFN significantly reduced mortality (OR, 13.5; 95% CI, 1.5–118). However, late administration did not reveal significant effects (OR, 2.1; 95% CI, 0.48–9.6) [36].

More recently, data from the WHO SOLIDARITY trial showed different findings [8, 9]. The SOLIDARITY RCT is an international, open-label, adaptive randomized trial of hospitalized COVID-19 patients. The trial included an IFN treatment arm. The regimen for IFN (mainly subcutaneous) was three doses (44 μg/dose) over a period of 6- days. The trial was conducted at 405 hospitals in 30 countries and 2063 were allocated to receive IFN (including 651 to IFN plus LPV/r). Death occurred in 243 of 2050 patients receiving IFN and in 216 of 2050 in the control group (risk ratio, 1.16; 95% CI, 0.96–1.39; P = 0.11). Nevertheless, subgroup analyses suggested that IFN-based therapy was associated with higher mortality in ventilated vs. non ventilated patients and in patients started on treatment >2 days after admission as compared to earlier initiation of therapy [9].

On the other hand, FPV has not been extensively examined in RCTs. FPV is a purine nucleoside analogue and a virus RNA-dependent RNA polymerase inhibitor that was originally approved in 2014 by the Japan Pharmaceuticals and Medical Devices Agency for influenza A virus infection treatment. It works as a chain terminator at the site of incorporation of the viral RNA thus decreasing the viral load. It is not toxic to mammalian cells because it does not inhibit their RNA or DNA synthesis [37].

In a small open-label non-randomized before-and-after study by Cai et al., clinical outcome of 80 adult patients with non-severe COVID-19 was evaluated by comparing 2 groups: 35 in FPV arm versus 45 patients in the LPV/r arm. Both groups received aerosol inhalation of IFN-α 1b twice daily. Primary end points were time to viral clearance and improvement in chest CT by day 14. Patients on FPV displayed a shorter time to viral clearance (median of 4 days, IQR: 2.5–9) than LPV/r arm (median of 11 days, IQR: 8–133) (P < 0.001) [9].

Despite being included in multiple national treatment protocols for COVID-19, the evidence supporting FPV use remains weak. A recent systematic review by the EUnetHTA Rolling Collaborative Review (RCR11) Authoring Team [14] concluded that the current evidence base does not support the use of FPV as monotherapy or combination therapy for COVID-19. In another recently updated WHO living systematic review and network meta-analysis, FPV was found to have no effect on mortality with an absolute risk reduction of -41 per 10000 (-113 to 207). The 95% credible interval included no effect, and certainty of evidence was rated as very low [38].

### Strengths and limitations

Our study has multiple strengths. First, to our knowledge, this is the first comparative study of IFN-based therapy vs. FPV in COVID-19. Second, although observational in nature, our study is less susceptible to treatment selection bias given that the allocation to treatment groups was based on drug availability and national treatment protocols which minimizes the risk of confounding by indication. Third, our analysis accounted for immortal time bias. Not considering the immortal time in the design or analysis of observational studies could lead to inflated treatments’ effect estimates. Based on data from at least one study in COVID-19 [39] and another influenza study [40], mortality relative risk increased by up to 60% when not considered treatment as a time-dependent variable in the Cox regression analysis [38]. However, our study has several limitations. First, our study was not an RCT and therefore we cannot completely rule out the effect of unmeasured or residual confounding or treatment selection bias. Second, the low number of events limited our ability to adjust for many potential confounders in the Cox model. Third, because our protocol did not allow the use of IFN-based therapy 7 days after symptoms onset, we could not compare the effect of early vs. late IFN effect on outcomes. Fourth, our study is a single center experience. Fifth, although, prehospital treatment might affect patients’ outcomes [41], we believe that this it is unlikely to affect our findings as in this study the majority of patients received either no prehospital therapy due to unavailability of effective antiviral therapy for COVID-19 in the outpatient setting at the date of the study, or very few received hydroxychloroquine in the outpatient setting. However, multiple RCTs have failed to show any benefit of hydroxychloroquine on all outcomes [42]. Moreover, If any prehospital effect is present, it should cause a random error rather than bias. Additionally, we have considered treatment with IFN- based therapy and FPV as time-dependent variables and adjusted for time since symptoms onset to address any raised survivor bias [23, 25]. Finally, patients with respiratory failure requiring mechanical ventilation, ARDS, and patients with CRS were excluded from our study, which limits the generalizability of our findings to critically ill patients.

## Conclusions

In this quasi-experimental comparative analysis of hospitalized patients with non-critical COVID-19, we observed that early IFN-based triple therapy was associated with better outcomes as compared to FPV with a statistically significant reduction in mortality at 28 days and a reduced need for adjunctive systemic corticosteroids. Future randomized controlled trials are needed to compare these 2 treatment regimens.

## Supporting information

S1 Table COVID-19 patient assessment form. (PDF)

S2 Table Primary outcome of mortality at 28 days. (PDF)

S3 Table Secondary outcome of mechanical ventilation. (PDF)

S4 Table Secondary outcome of intensive care unit admission. (PDF)

S5 Table Adverse events of interferon-based triple therapy vs. favipiravir. (PDF)

S1 File Study raw data. (SAV)
