# Supplementary material for: Combination of (interferon beta-1b, lopinavir/ritonavir and ribavirin) versus favipiravir in hospitalized patients with non-critical COVID-19: A cohort study
Source: PLoS One. 2021 Jun 10;16(6):e0252984. doi: 10.1371/journal.pone.0252984 (PMC8191942; doi:10.1371/journal.pone.0252984)
Supplement: S1 Table — (PDF) [file pone.0252984.s001.pdf]

وزارة الصحة
مدينة الملك فهد الطبية
King Fahad Medical City

COVID-19 Patient Assessment Form

Patient's Addressograph

MARK ☒ All THAT APPLY:

- ☐ COVID-19 **Suspected** Case
- ☐ COVID-19 **PCR Confirmed** Case
- ☐ New Case
- ☐ Follow-up Case
- ☐ ICU
- ☐ ED
- ☐ Ward

Allergies	
Age	
Duration of symptoms	
**High Risk for Severe Disease**	
☐ CAD ☐ CHF ☐ CKD<br>☐ DM ☐ COPD<br>☐ Asthma ☐ Obesity<br>☐ Immunocompromised<br>☐ Hypertension<br>☐ Other------------------------------	
**Laboratory Studies**	
Ferritin	
CRP	
D-dimer	
LDH	
Creatinine	
CK	
LFTs	
WBC	
ALC	
ANC	
Hb	
Platelet	
**QTc interval**	
QT prolonging agents	
Chest X-ray	☐ clear<br>☐ new infiltrates<br>☐ stable<br>☐ worsening
Other workup	

Current Treatment (days)*

- ☐ 1 [Triple antiviral] (---------)
- ☐ 2 [Ribavirin/Kaletra] (---------)
- ☐ 3 [Tocilizumab] (---------)
- ☐ 4 [Steroids] (---------)
- ☐ 5 [C. Plasma] (---------)
- ☐ 6 [Favipiravir] (---------)

Progress:

☐ **Mechanical ventilation**

SOB (non-intubated):

- ☐ **I**mproving **[I]**
- ☐ **S**table **[S]**
- ☐ **W**orsening **[W]**

Oxygenation:

☐ I ☐ S ☐ W

FIO$_2$: --------------------

Temperature: ---------------------

Hemodynamics:

☐ I ☐ S ☐ W

Concomitant Infections:

- ☐ HAP/VAP
- ☐ UTI
- ☐ Bacteremia
- ☐ ------------------

Positive cultures:

- ☐ ----------------------
- ☐ -----------------------

Respiratory Viral Panel:

- ☐ Positive
- ☐ Negative

Current antibiotics (day)

- ☐ Meropenem (---------)
- ☐ Tazocin (---------)
- ☐ Vancomycin (---------)
- ☐ Azithromycin (---------)
- ☐ Other (---------)

Other supportive therapeutics:

- ☐ Statins (---------)
- ☐ Zinc (---------)
- ☐ Vitamin D (---------)
- ☐ Thromboprophylaxis (---------)
- ☐ Other (---------)

Disease Classification*

- ☐ Mild
- ☐ Moderate
- ☐ Severe
- ☐ Critical
- ☐ With Cytokine Release Syndrome

Complications:

- ☐ ARDS
- ☐ MSOF
- ☐ PE
- ☐ VTE
- ☐ Dialysis

Patient's Assessment:

☐ I ☐ S ☐ W

Recommendations:

1. Thromboprophylaxis: Follow KFMC COVID-19 protocol
2. Start regimen (see order sheet for details)
   - ☐ 1 [Triple antiviral]
   - ☐ 2 [Ribavirin/Kaletra]
   - ☐ 3 [Tocilizumab]
   - ☐ 4 [Steroids]
   - ☐ 5 [C. Plasma]
   - ☐ 6 [Favipiravir]
3. Continue regimen
   - ☐ 1 [Triple antiviral]
   - ☐ 2 [Ribavirin/Kaletra]
   - ☐ 3 [Tocilizumab]
   - ☐ 4 [Steroids]
   - ☐ 5 [C. Plasma]
   - ☐ 6 [Favipiravir]
4. Discontinue regimen
   - ☐ 1 [Triple antiviral]
   - ☐ 2 [Ribavirin/Kaletra]
   - ☐ 3 [Tocilizumab]
   - ☐ 4 [Steroids]
   - ☐ 5 [C. Plasma]
   - ☐ 6 [Favipiravir]
5. ☐ Repeat COVID-19 PCR
6. Other recommendations:

Abbreviations:

CAD: Coronary artery disease<br>DM: Diabetes mellitus<br>CHF: Congestive heart failure<br>COPD: Chronic Obstructive Pulmonary Disease<br>CKD: Chronic Kidney Disease<br>MSOF: Multisystem organ failure	LFT: Liver function test<br>WBC: White blood cell count<br>ALC: Absolute lymphocyte count<br>ANC: Absolute neutrophil count<br>Hb: Hemoglobin<br>HAP: Hospital acquired pneumonia<br>Tazocin: Piperacillin/Tazobactam<br>Kaltera: lopinavir/ritonavir	VAP: Ventilator associated pneumonia<br>VTE: Venous thromboembolism<br>UTI: Urinary tract infection<br>I: Improving<br>S: Stable<br>W: Worsening<br>PE: Pulmonary embolism

**Physician's Name/Stamp**		**Physician's Signature**		**Date/Time**	

Please refer to Second Health Cluster in Central Region ***Coronavirus Disease 2019 (COVID-19) Management Guideline – Version 1.4*** *(posted at the COVID-19 Link on KFMC Intranet Main Webpage) for full guidance – the table below is an extracted summary of important points from the guideline.*

**TREATMENT RECOMMENDATIONS BASED ON CLINICAL CATEGORY AND DISEASE SEVERITY**		
**Clinical Category**	**Respiratory Status**	**Treatment Recommendations for *Suspected OR Confirmed* COVID19**
**All Adult hospitalized patients**	Any	☐ Statins when indicated for hypercholestrolemia or for CAD<br>☐ Thromboprophylaxis per COVID-19 protocol
**Mild**	- Flu-like Symptoms: Fever, sore throat, rhinorrhea, cough, myalgia<br>- GI Symptoms, loss of smell or taste<br>- **No shortness of breath or O2 requirements** and **no radiologic evidence of pneumonia**	☐ **If symptom onset was < 7 days:** triple combination therapy with Interferon β-1b+ Lopinavir/ritonavir+ ribavirin<br>☐ **OR** Favipiravir
**Moderate**	- Shortness of breath and/or chest imaging consistant with pneumonia.<br>- Blood oxygen saturation <90-93% responding to 1-3 L of O2 by nasal canula or no change from baseline respiratory support requirement	☐ **If symptom onset was < 7 days:** triple combination therapy with Interferon β-1b+ Lopinavir/ritonavir+ ribavirin<br>☐ **OR** Favipiravir<br>☐ Antibiotic therapy if bacterial pneumonia is of a concern:<br>○ CAP: Ceftriaxone +/- Vancomycin +/- Azithromycin[†]<br>○ HAP: Cefepime +/- Vancomycin
**Severe**	Shortness of breath and/or chest imaging consistant with pneumonia **PLUS** ≥ 1 of the following symptoms:<br>○ Respiratory rate≥30/min<br>○ Blood oxygen saturation ≤93%<br>○ PaO2/FiO2 ratio <300<br>○ Lung infiltrates >50% of the lung field within 24-48 hours	☐ Convalescent plasma transfusion[§]<br>☐ Favipiravir (or Remdesivir when available)<br>☐ OR may lopinavir/ritonavir+ ribavirin<br>☐ Antibiotic therapy if bacterial pneumonia is of a concern:<br>○ CAP: Ceftriaxone +/- Vancomycin +/- Azithromycin[†]<br>○ HAP: Cefepime +/- Vancomycin<br>☐ Dexamethasone or Methylprednisolone may be used if patient has the criteria below
**Critical**	Respiratory failure requiring mechanical ventilation, acute respiraotry distress syndrome (ARDS), multi-organ failure, sepsis, altered consciousness, patient with cytokine release syndrome	☐ Convalescent plasma transfusion[§]<br>☐ Favipiravir (or Remdesivir when available)<br>☐ OR lopinavir/ritonavir+ ribavirin<br>☐ Antibiotic therapy if bacterial pneumonia is of a concern:<br>○ CAP: Ceftiraxone +/- Vancomycin +/- Azithromycin[†]<br>○ HAP: Cefepime +/- Vancomycin<br>☐ If cytokine release syndrome is confirmed or patient has high risk of developing it, consider starting Tocilizumab<br>☐ Dexamethasone/Methylprednisolone may be used if patient has the criteria below

Consideration for steroids:

- ☐ Moderate disease with new supplemental oxygen ≥ 4 L or escalating oxygen requirements from baseline
- ☐ Severe disease and mechanically ventilated patients with ARDS

Criteria for patients at high risk of developing cytokine release storm (consideration for tolicuzimab):

Confirmed COVID-19 AND Chest X-ray consistent with COVID-19 pneumonia with rapidly worsening respiratory symptoms/signs AND absence of systemic bacterial or fungal coinfection PLUS 2 or more of the following:

- Ferritin >300 ug/L with doubling within 24 hours
- Ferritin >600 ug/L at presentation
- LDH >250
- Elevated D-dimer (>1 mcg/mL)
- CRP > 70 mg/L

Tocilizumab is associated with a higher risk of secondary bacterial and fungal infections

Reduce Lopinavir/ritonavir to QD IF:

- ☐ *QTc>480 msec*
- ☐ *Patients <480 msec with:*
  - ☐ *First-degree or bundle branch block*
  - ☐ *Bradycardia*
- ☐ *ALT more than three times the upper limit of normal*

Check drug interactions with Favipiravir and Lopinavir/ritonavir

[†] Re-evaluete the indication for using azithromycin if will be administered in concomitant with other QTc prolonging agents.

[§] To enroll your patient, please contact Dr. Nawal Alshehry, the principal investigator of the clinical trial, at: nalshehry@kfmc.med.sa (0558983336)
