# Supplementary material for: Combination of (interferon beta-1b, lopinavir/ritonavir and ribavirin) versus favipiravir in hospitalized patients with non-critical COVID-19: A cohort study
Source: PLoS One. 2021 Jun 10;16(6):e0252984. doi: 10.1371/journal.pone.0252984 (PMC8191942; doi:10.1371/journal.pone.0252984)
Supplement: S2 Table — (PDF) [file pone.0252984.s002.pdf]

**S2 Table. Primary outcome of mortality at 28 days.**

| Variable | Model 1 | Model 2 |
|---|---|---|
| Triple combination therapy, HR (95% CI) | 1.00 (0.57-1.76) | 0.27 (0.08-0.88) |
| C statistic* | 0.722 | 0.859 |
| Akaike Information Criterion* | 198.5 | 90.4 |
| *P* value | 0.182 | 0.005 |

Model 1; Patient baseline variables at admission; Age, Male sex, body mass index, and $SpO_2$.
Model 2; Patient COVID-19 related severity variables during hospital stay; serum CRP, ferritin, D-Dimer, and bilirubin levels, WBC, oxygen needs and systemic use of dexamethasone.
Immortal time bias was accounted for in the Cox proportional models as triple combination therapy was as time-dependent variables.
*Higher values for C statistic and lower values for Akaike Information Criterion indicate better models.
