# Supplementary material for: Combination of (interferon beta-1b, lopinavir/ritonavir and ribavirin) versus favipiravir in hospitalized patients with non-critical COVID-19: A cohort study
Source: PLoS One. 2021 Jun 10;16(6):e0252984. doi: 10.1371/journal.pone.0252984 (PMC8191942; doi:10.1371/journal.pone.0252984)
Supplement: S3 Table — (PDF) [file pone.0252984.s003.pdf]

S3 Table. Secondary outcome of mechanical ventilation.

Variable	Model 1	Model 2
Triple combination therapy, HR (95% CI)	1.27 (0.88-1.84)	1.22 (0.68-2.20)
C statistic*	0.322	0.538
Akaike Information Criterion*	378.1	207.4
P value	0.076	0.027

Model 1; Patient baseline variables at admission; Age, Male sex, body mass index, and $SpO2$.
Model 2; Patient COVID-19 related severity variables during hospital stay; serum CRP, ferritin, D-Dimer, and bilirubin levels, WBC, oxygen needs and systemic use of dexamethasone.
Immortal time bias was accounted for in the Cox proportional models as triple combination therapy was as time-dependent variables.
*Higher values for C statistic and lower values for Akaike Information Criterion indicate better models.
