# Supplementary material for: Combination of (interferon beta-1b, lopinavir/ritonavir and ribavirin) versus favipiravir in hospitalized patients with non-critical COVID-19: A cohort study
Source: PLoS One. 2021 Jun 10;16(6):e0252984. doi: 10.1371/journal.pone.0252984 (PMC8191942; doi:10.1371/journal.pone.0252984)
Supplement: S5 Table — (PDF) [file pone.0252984.s005.pdf]

S5 Table. Adverse events of interferon-based triple therapy vs. favipiravir.

Variables	Triple therapy (n=68)	Favipiravir (n=154)	*P-value*
Nausea	9 (13)	5 (3)	0.013
Diarrhea	12 (18)	5 (3)	<0.0001
Hemoglobin (11-16 g/dL)			
Baseline	13.6 (12-14.3)	13.5 (12.1-14.7)	0.995
Day 3	13.3 (12-14.7)	13.1 (11.6-14.3)	0.324
Day 7	13.7 (12.5-14.8)	13.1 (11.5-14.8)	0.131
Platelets (115-435 x 10^9 per L)			
Baseline	208 (155.5-298.5)	229 (173-284)	0.562
Day 3	307 (219-455)	287 (231-398)	0.364
Day 7	374 (271-458)	370 (279-465)	0.727
Alanine transaminase (0-55 U/L)			
Baseline	35 (25-60)	37 (24-60.5)	0.849
Day 3	54 (31-80.5)	43 (30.5-78.5)	0.508
Day 7	63 (37-116)	55 (38-91)	0.406
Bilirubin (3-20 µmol/L)			
Baseline	4.9 (3.7-7.6)	4.4 (3.3-6.4)	0.283
Day 3	6.5 (4.1-9.1)	4.3 (3.3-6.3)	0.001
Day 7	5.9 (4-7.8)	4.1 (3.1-5.7)	0.001

Data are n (%) or median (IQR). In the triple therapy group 68 patients were treated with triple combination of interferon beta-1b, lopinavir–ritonavir, and ribavirin. U/L=units per L.
